# Supplementary material for: A spider silk-derived solubility domain inhibits nuclear and cytosolic protein aggregation in human cells
Source: Commun Biol. 2022 May 26;5:505. doi: 10.1038/s42003-022-03442-5 (PMC9135726; doi:10.1038/s42003-022-03442-5)

Supplementary Figure 1

Hela cells were transiently transfected with AgDD-sfGFP or NT*-sfGFP and incubated in the absence or presence of 1 μM Shield-1 for 16 hours. Cells were fixed 24 hrs after transfection with 4% PFA and counterstained with Hoechst. Scale bar 20 μm.

Supplementary Figure 1

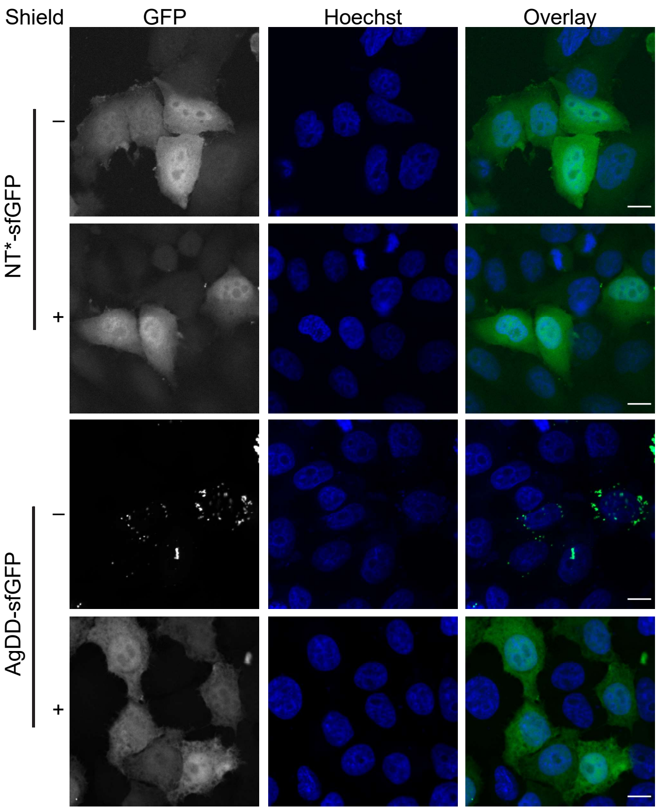

Supplementary Figure 2

Uncropped scans of western blots. Labels refer to the panels of the figures in the main manuscript.

Figure 1b

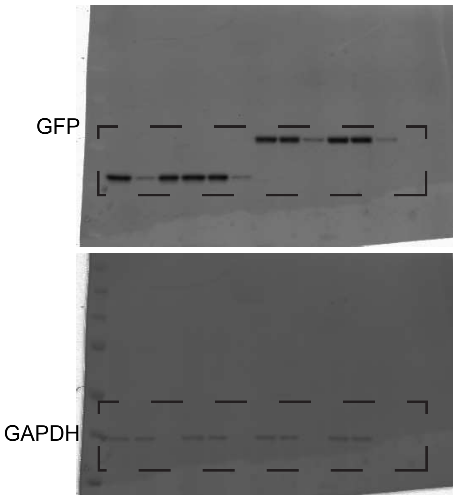

Figure 2c

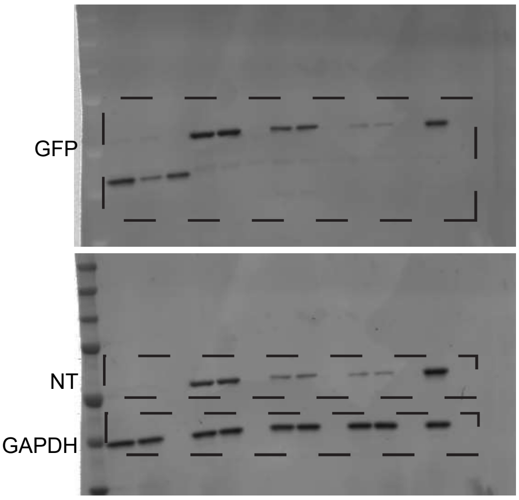

Figure 3c

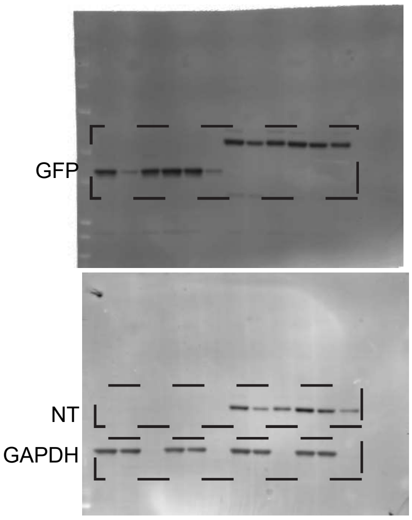

Figure 3d

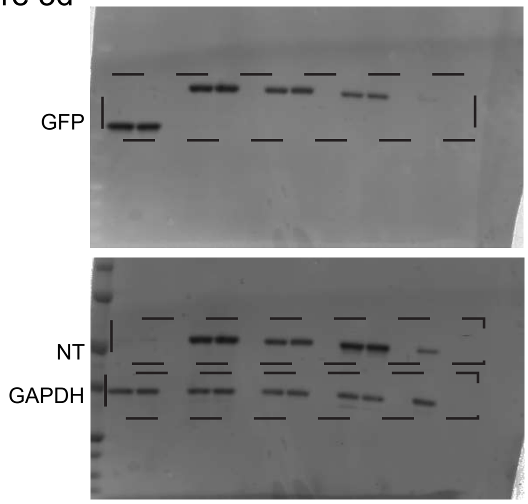

Supplement: Supplementary file 2 — Supplementary Information [file 42003_2022_3442_MOESM2_ESM.pdf]
