# Supplementary material for: A spider silk-derived solubility domain inhibits nuclear and cytosolic protein aggregation in human cells
Source: Commun Biol. 2022 May 26;5:505. doi: 10.1038/s42003-022-03442-5 (PMC9135726; doi:10.1038/s42003-022-03442-5)
Supplement: Supplementary file 3 — Description of Additional Supplementary Files [file 42003_2022_3442_MOESM3_ESM.pdf]

Description of Additional Supplementary Files

File name: Supplementary Data 1
Description: The original data of the graphs.
